# The role of community pharmacists and pharmacies in physical activity promotion: an interdisciplinary e-Delphi study

**DOI:** 10.1007/s11096-024-01731-z

**Published:** 2024-04-25

**Authors:** Ruben Viegas, Mara Pereira Guerreiro, Filipa Duarte-Ramos, Romeu Mendes, Filipa Alves da Costa

**Affiliations:** 1 Faculty of Pharmacy, University of Lisbon – Imed, Research Institute for Medicines, Av. Prof. Gama Pinto, 1649-003 Lisbon, Portugal; 2 Egas Moniz Center for Interdisciplinary Research (CiiEM), Egas Moniz School of Health and Science, Caparica, Portugal; 3 EPIUnit - Instituto de Saúde Pública, Universidade do Porto, Porto, Portugal; 4 Laboratório para a Investigação Integrativa e Translacional em Saúde Populacional (ITR), Porto, Portugal; 5 Unidade Local de Saúde de Trás-os-Montes e Alto Douro, Vila Real, Portugal

**Keywords:** Community pharmacy services, Consensus development, Healthcare workforce, Health promotion, Physical activity, Primary health care

## Abstract

**Background:**

Physical activity has a key role in the prevention and control of noncommunicable diseases. Community pharmacists are an accessible source to provide brief advice to people on how to be more physically active. Nonetheless, there is a limited understanding of stakeholders' perspectives on their role in promoting physical activity, to inform policy and practice. The present study addresses this gap.

**Aim:**

To determine consensus from different health professionals on the role of pharmacists and pharmacies in brief physical activity counselling in Portugal.

**Method:**

This cross-sectional study used a two-round e-Delphi panel. The questionnaire was organised into four domains of physical activity promotion and comprised 37 items. Interdisciplinary experts rated their level of agreement using a 5-point Likert scale. Consensus was set at the outset as 75% or more of participants scoring 4 or 5 (consensus “in”) or 1 or 2 (consensus “out”).

**Results:**

Forty-two health professionals involved in promoting physical activity in the ambulatory setting in Portugal were selected through purposive quota sampling. Eighteen out of 37 items were consensual in the first round and five more achieved consensus after the second round (62.2%). Physical activity promotion was seen as the role of all healthcare workforce and pharmacies were considered as suitable spaces for service provision, regardless of remuneration.

**Conclusion:**

This study endorses a set of roles for physical activity promotion in community pharmacy from an interdisciplinary perspective. Consensually established perspectives can inform policy making and practice, streamlining the coordination of pharmacies with the national health service.

**Supplementary Information:**

The online version contains supplementary material available at 10.1007/s11096-024-01731-z.

## Impact statements

- Consensual interprofessional perspectives on the role of community pharmacists and pharmacies on physical activity promotion offer a foundation for the development of policies and guidelines more likely to gain support from healthcare stakeholders.
- Community pharmacies are perceived as suitable spaces for physical activity promotion within primary care.
- Elderly, people living with diabetes or with depressive disorders may be priority groups for pharmacists’ physical activity promotion.
- By reaching consensus, different professionals can have a unified voice about physical activity promotion and thus inform policy.

## Introduction

Physical activity is one a main contributor to improved health, considered by the World Health Organization (WHO) a ‘best buy’ for governments to tackle non-communicable diseases and improve health outcomes [1]. Even limited physical activity can positively impact health outcomes, especially in sedentary individuals. Targeting sedentary time can improve health outcomes for different health conditions, including type 2 diabetes and cardiovascular conditions [2].

Physical activity has a wide range of benefits to the human body, like the reduction of fat mass, improvements in blood pressure and heart rate, and better glucose uptake [3]. These benefits are important for the general population, and for many different clinical populations [4].

Healthcare professionals have opportunities to promote physical activity as they are in frequent contact with individuals and can engage in conversations on how to be more active. Despite these opportunities, the lack of interdisciplinary approaches hinders the implementation of physical activity promotion across the healthcare system [5].

To integrate physical activity promotion in the person’s journey across the healthcare system, additional efforts should be put into overcoming barriers like limited training for healthcare professionals and investments in awareness raising in the population [6–8]. The Portuguese General Health Directorate (DGS) developed the strategy to tackle growing sedentary behaviour and obesity, supported by tools to promote physical activity, mostly targeting primary care [9]. One of these is a free training course, directed at all healthcare professionals, to provide them with the necessary skills and competences to reinforce health promotion in this area. Despite the National Health Plan 2021–2030 underscoring the importance of leveraging community pharmacies for enhanced public health promotion, no attention has been given to their integration in physical activity promotion efforts [10].

Community pharmacists can act as a gateway to support lifestyle choices by providing a range of services, some of which are already provided, like smoking cessation. Other preventive services also exist, including immunisation and HIV and viral hepatitis testing, to name a few. Previous research suggested that community pharmacists are motivated to engage in physical activity promotion, despite stressing that training and structured processes are needed [11]. Several studies have investigated community pharmacists’ interventions in physical activity promotion, although few reported robust outcomes [12]. Research exploring stakeholders' perspectives on pharmacists’ input into strengthening physical activity is needed.

### Aim

This study aimed to determine consensus from different health professionals on the role of pharmacists and pharmacies in physical activity promotion in Portugal.

### Ethics approval

Ethical approval was granted by the Ethics Committee of the Faculty of Pharmacy of the University of Lisbon (#06/2021, October). Written informed consent was obtained from all participants.

## Method

### Research design

A cross-sectional study was used, where consensus was sought between February and May 2023 using an online Delphi panel. The study protocol was not previously registered but the number of rounds was defined a priori as 2 rounds. Consensus was set a priori for both rounds as ≥ 75% of participants scoring 4 or 5 (consensus “in”) or 1 or 2 (consensus “out”) [13].

### Sample and recruitment

Delphi studies rely on experts [14]; here defined as a person practising in primary care in Portugal (outpatients), with experience in brief advice or other forms of physical activity counselling, and professional qualification, evidenced by a specialisation or comparable academic qualification. An inclusive approach was taken, by engaging health professionals involved in the promotion of physical activity promotion, disease prevention and self-management support in the outpatient health care setting. As there is no ultimate guidance for the dimension of a Delphi panel, the target was to include five participants in each professional category, totalling 35 participants (quota sampling), to secure a spectrum of opinions and diversity in the panel [15].

An invitation to participate in the study was emailed to the regulatory bodies for nurses, nutritionists, pharmacists, physicians and psychologists, asking support in dissemination. The call included a flyer with a link to opt into the study by filling the informed consent (operationalised using a Google Form, where contact details and demographic data were requested). Additional experts were sought by direct contacts from the research team to increase participation. Panellists and those declining to participate were asked to forward the invitation or suggest other contacts with a similar professional status (snowballing sample). Experts were recruited without limitations to the geographical residence or place of work in Portugal and assessed against the eligibility criteria. Consenting participants were assigned a code used to track their responses among rounds and ensure quasi-anonymity. The identity of panellists was concealed from one another, but it was known to the main researcher (RV).

### Questionnaire development

A literature review was used to identify key topics, further supplemented by insights from previous work [11, 12] and the professional experience of the research team (possessing expertise in both physical activity promotion, consensus and mixed methods) resulting in the first-round questionnaire. The most significant issues covered were organised into four domains and addressed issues of remuneration for service provision and training for pharmacists: (1) Importance of promoting physical activity in the community (6 questions), (2) Pharmacists as physical activity promotors (7 questions), (3) Pharmacies as settings to promote physical activity (8 questions), and (4) Opportunities for pharmacists’ interventions (16 questions).

Statements were presented in each domain, and agreement requested using a 5-point Likert scale (1—completely disagree to 5—completely agree). In the first round of this Delphi panel, a free-text field enabled suggestions. In both rounds a box after each domain allowed additional comments.

Professional field and specialization, work setting, gender, population covered (urban, rural or mixed) and years of experience were also collected. The initial version of the e-Delphi questionnaire was pre-tested via cognitive interviews [16] with five experts, including a pharmacist, a physician, a nurse, an exercise physiologist and a nutritionist. They assessed the clarity and organisation of the statements. The interview transcripts and notes were analysed resulting in a modified version (Supplementary file 1). Due to the difficulty in recruiting participants and no apparent conflict in their participation these experts were also invited to the main rounds.

### Data collection

The survey was made available online through a link sent to the email provided in the informed consent form – one form for each round. Data were extracted into an Excel spreadsheet for data analysis. Each round was open for 35 days, during which 3 reminders (at 15, 21 and 30 days) were sent. Participants were also contacted personally via text message in case of no reply to reminders. Participants were not provided any additional background information before the first round but had access to relevant links such as legislation or research papers through the Google Form questionnaire. There was no compensation for participation in the study, but a certificate of participation was provided for those completing both rounds.

After the first round, participants received their responses, the group median and a summary of the comments so they could reply to the second round.

### Data management and analysis

Quantitative data from the closed-ended open questions were analysed by independent researchers to ensure validity (RV, FAC). Data values for each round are presented as percentage of consensus “in” for each of the items. All data were stored on a password-protected device and carefully managed assuring data confidentiality. The reporting guideline ACCORD for consensus methods in biomedicine developed via a modified Delphi, that focuses specifically in quality assessment of these studies was used to report this study [17].

## Results

There were 42 professionals signing the informed consent (15 recruited through regulatory bodies and 27 through the mailing list), although only 30 participated in the first round (71.4%). There were 23 completing the second round (76.7%). Professional and demographic characteristics are presented in Table 1.

**Table 1:** Initial sample participant’s characteristics

	Completed first round (n = 30), n (%)	Completed second round (n = 23), n (%)
*Gender*		
Male	8 (26.7)	6 (26.1)
Female	22 (73.3)	17 (73.9)
*Professional category*		
Exercise and health	4 (13)	2 (9)
Medicine	6 (20)	5 (22)
Nursing	3 (10)	1 (4)
Nutrition	1 (3)	1 (4)
Pharmaceutical sciences	11 (37)	9 (39)
Physiotherapy	1 (3)	1 (4)
Psychology	4 (13)	4 (17)
*Specialization*		
Community pharmacy	10 (33)	8 (35)
Hospital pharmacy	0 (0)	0 (0)
Rheumatology	1 (3)	1 (4)
General and family medicine	4 (13)	3 (13)
General nursing	1 (3)	0 (0)
Community nursing	1 (3)	0 (0)
Clinical nutrition	1 (3)	1 (4)
Clinical and health psychology	3 (10)	3 (13)
Educational psychology	0 (0)	0 (0)
Physical activity and health	4 (13)	2 (9)
N/A	5 (17)	5 (22)
*Work setting*		
Primary care	23 (77)	19 (83)
Other settings	7 (23)	4 (17)

Eighteen out of 37 items (48.7%) reached consensus on the first round and another five on the second round. Results of both rounds are presented in Table 2 and free-text field comments from both rounds available as supplementary file 2.

**Table 2:** Consensus for both rounds

Domain	Item	Number people expressing agreement as 4 or 5/number of participants (% consensus) in R1	Number people expressing agreement as 4 or 5/number of participants (% consensus) in R2	Round where consensus was reached
1	Promoting physical activity should be part of the role of all health professionals, especially those working in primary health care	30/30 (100)	23/23 (100)	R1
1	It is important that community health workers assess and record physical activity indicators such as number of steps, minutes of physical activity, intensity, and sedentary time, among others	27/30 (90)	15/23 (65)	R1
1	It is important that in the community health professionals provide brief physical activity advice to their users	29/30 (97)	23/23 (100)	R1
1	Physical activity promotion should be included as a pre-service training module for different health professionals	26/30 (87)	21/23 (91)	R1
1	Physical activity promotion should be included as a postgraduate training module for different health professionals	22/30 (73)	20/23 (87)	R2
1	There is a need to include physical activity promotion in the policy agenda of health promotion by health professionals	28/30 (93)	23/23 (100)	R1
2	Ordinance 97/2018 expands the services that pharmacies can provide and indicates that "Pharmacies may also promote campaigns and programmes for health literacy, disease prevention and promotion of healthy lifestyles." The role of the community pharmacist in promoting physical activity falls under this ordinance	26/30 (87)	22/23 (96)	R1
2	Decree-law 62/2016 indicates that “The Ministry of Health may contract with community pharmacies, in their areas of competence, the provision of public health intervention services framed in the priorities of health policy, namely programmes integrated with primary health care”. The promotion of physical activity can be framed within the services described in the decree-law	21/30 (70)	20/23 (87)	R2
2	The National Plan for Physical Activity Promotion of the Directorate General of Health aims to "Train health professionals and promote structural and functional changes in order to generalize the promotion of physical activity in health services". Pharmacists need to have specific training on physical activity promotion to be able to competently provide this type of service	24/3080)	18/23 (78)	R1
2	The pharmacist may have an important role in referring users to places or resources in the community where they can be more physically active	29/30 (97)	20/23 (87)	R1
3	Community pharmacies are suitable spaces for physical activity promotion	21/30 (70)	20/23 (87)	R2
3	Pharmacies should be more involved in organising physical activity promotion activities, such as walking groups	21/30 (70)	21/23 (91)	R2
3	Similar to what already happens with nutrition consultations, pharmacies could also have a differentiated consultation with an exercise professional	26/30 (87)	18/23 (78)	R1
3	Within 10 years it is expected that pharmacies could be considered suitable venues for physical activity promotion	16/30 (53)	19/23 (83)	R2
4	Promoting physical activity in pharmacies can be especially important in people living with the following diseases: Type 2 diabetes	26/30 (87)	19/23 (83)	R1
4	Promoting physical activity in pharmacies can be especially important in people living with the following conditions: Depressive disorder	27/30 (90)	20/23 (87)	R1
4	Promoting physical activity in pharmacies can be especially important in people living with the following conditions: Anxiety disorders	25/30 (83)	19/23 (83)	R1
4	Promoting physical activity in pharmacies can be especially important in people living with the following diseases: Metabolic syndrome	23/30 (77)	19/23 (83)	R1
4	The promotion of physical activity in pharmacies can be especially important for the following population groups: Elderly (> 65 years)	27/30 (90)	21/23 (91)	R1
4	Promoting physical activity in pharmacies can be especially important for the following population groups: Smokers	25/30 (83)	19/23 (83)	R1
4	Promoting physical activity in pharmacies can be especially important for the following population groups: Obese people (BMI > 30)	27/30 (90)	21/23 (91)	R1
4	Promoting physical activity in pharmacies can be especially important for the following population groups: People living in social isolation	27/30 (90)	20/23 (87)	R1
4	Promoting physical activity in pharmacies can be especially important for the following population groups: People at high risk of chronic disease (e.g. diabetes, people with risk factors for cardiovascular disease)	27/30 (90)	19/23 (83)	R1

There were 14 items not reaching consensus after the second round. The items dropped were:

- Some countries have a model of "health champions" who are individuals with specific training to advise people to adopt a healthier lifestyle. Pharmacists can adapt this model and be 'health champions' in promoting physical activity in Portugal.
- There is a need for the regulator of the pharmacy profession to create a new specific competence, which is not yet legislated, for the promotion of physical activity by pharmacists.
- Individual physical activity promotion may be the most important role the pharmacist can play in promoting physical activity.
- Pharmacy should be remunerated for brief advice on physical activity by the NHS; and by users (2 statements).
- The current remuneration model focused on the sale of drugs and health products from pharmacies is a barrier to implementing these types of services in the future.
- Pharmacies' computer systems should be able to record the physical activity levels of their users.
- Promoting physical activity in pharmacies can be especially important in people living with musculoskeletal pain; chronic obstructive pulmonary disease; rheumatoid arthritis; angina; heart failure; pregnant women; and adolescents (7 statements).

## Discussion

### Statement of key findings

Physical activity promotion was consensually perceived as part of the role of all healthcare professionals, especially those working in primary health care. Community pharmacists, as an integrated profession in primary care were perceived as a partner for physical activity promotion in Portugal, through an interdisciplinary approach and thus consolidating their role as health promotors.

### Strengths and weaknesses

This study’s strengths include the interdisciplinary approach to explore perspectives about physical activity promotion through pharmacies. The stability of consensus reached across both rounds attests the strength of our work. This study adds insights to the work currently developed by the Portuguese physical activity promotion plan (DGS) that focuses on healthcare professionals fully integrated into the national health system. Limitations include selection bias as the mailing sent through professional societies was not successful in recruitment, leading us to use a mailing list of contacts. Between the first and second round of the Delphi, attrition occurred (45%). Moreover, there was a limited number of professionals from specific areas such as nutrition and physiotherapy. All these factors may impact the validity of the results and reinforces the notion that Delphi findings should be interpreted by recognizing that they might undergo refinement in light of future research.

### Interpretation

Most items focusing on the importance of physical activity as part of the “One health” approach achieved consensus, as expected [5, 18]. Health promotion in general and on physical activity in particular was consensually seen as an integral role of all healthcare professionals, above all those practicing in settings focusing on prevention, i.e., primary care. Community pharmacists are essential members of the workforce team geographically spread across the country, and thus can further support behaviour change if trained and equipped with the right tools [19]. The scope of practice of pharmacists varies widely across countries, depending on the workforce available, population needs and political will [20, 21]. It also changes throughout time and sometimes there are windows of opportunity that may accelerate change [22, 23].

Short and brief interventions (SBI) for addressing risk factors have been emerging and expanding in recent years. Indeed, these SBI were consensually considered relevant to be routinely promoted by all healthcare professionals, including pharmacists [24]. Other authors have previously referred to interprofessional collaboration as key to success in physical activity promotion [25]. Even though there are specific professionals with a degree in this area, like physiotherapists, these focus on treatment, whereas health promotion and SBI are recognised as most successful when various actors intervene reinforcing each others’ messages. This is the approach we consider most suitable for Portugal but may well be different elsewhere. WHO has defined task shifting as “the rational redistribution of tasks among health workforce teams”, from trained and qualified health workers to other health workers with shorter training to maximize the available health workforce [26]. This approach could be particularly relevant in countries with shortages of qualified staff to exclusively take this role. In the United Kingdom, for example, healthy living pharmacies have been introduced aiming to expand the benefits of public health to a wider population by involving community pharmacy staff [27] specifically trained to take on this role, showing that solutions may vary across countries. The population groups for whom the service was consensually agreed as beneficial included people living with diabetes (83% agreement), depressive disorders (87% agreement) and the elderly (91% agreement). These three subgroups, often overlapping, are increasingly growing, and community pharmacists will continue supporting these groups [28, 29]. Other population subgroups were not consensual, such as people living with COPD, heart conditions, pregnant women, and adolescents. It is likely that the exclusion of these subgroups results from cautiousness, as noted: “The promotion of physical activity in certain population groups or people with certain diseases should be done with great care and with caution about the risks inherent to inappropriate physical activity”. More training in behaviour change is needed to address the issues of fear of recommending physical activity to specific population groups [30]. Changes in curricular structure are difficult but needed whenever the scope of practice of the profession changes. Therefore, it will be important for academia and professional bodies to identify the best solution to provide pharmacists with the required competency [31].

An item that was not consensual was “Individual physical activity promotion may be the most important role the pharmacist can play in promoting physical activity”, perhaps because other roles are equally or more important (e.g., medication review) or because a public health approach is perceived as more impactful in this context compared to an individual approach, as highlighted: “I understand promotion as actions, interventions or activities such as counselling/recommendations/incentives, provision of health literacy tools related to the topic, organization of one-off or fixed activities”. Some interventions, such as the organization of walking groups were consensual. However, to date, these strategies are seldomly implemented, except on commemorative dates [32].

Some items not reaching consensus focused on the regulation of the pharmacy profession and the need for developing specific competences for service provision. This may result from the interprofessional nature of the panel, where there might have been limited knowledge about pharmacists’ regulations. It could also result from the belief that training is important but does not justify imposing a specific competence to be acquired. As pointed out, “The pharmacist can be an important professional in promoting individual physical activity*,* but he/she must receive adequate training for that”. Pharmacies in Portugal can already promote campaigns and health literacy programmes that focus on disease prevention and health promotion by normative 97/2018 [33], but no clear reference is made to physical activity. While a new competency might not be needed, it has been suggested that more focus should be put into defining general competencies for health promotion strategies where pharmacists can contribute [34].

The remuneration around service provision was also not consensual. On one hand, the current remuneration model was not perceived as a barrier for service provision, neither was remuneration considered essential for implementation. One participant stated that “Professional work should be remunerated, by whom?…it will depend on the context probably. Ideally the NHS but we know that may not be a priority at present”. Previous studies suggest service remuneration is important to booster implementation [35]. However, many such studies fail to include the perspectives of other healthcare professionals. Furthermore, pharmacists themselves may perceive service remuneration differently according to service complexity, as stressed: “I believe that brief advice on physical activity should be an integral part of the provision of information to the patient and therefore should not be remunerated. This type of information should be provided to the patient at every contact. A differentiated service in physical activity that includes a consultation should be remunerated”.

Another aspect that did not reach agreement was the pharmacies’ computer systems being used to record physical activity levels. Although it is not current practice to measure physical activity levels in Portugal, some work [36] showed that using widely available activity trackers (smartphones or watches/bracelets) can provide important information. Technology can support monitoring and provide people living with chronic conditions additional clues on healthy behaviours [37].

Pharmacists’ involvement in health promotion is embedded in the legislation, so we consider the pharmacist should be supporting behaviour change by providing SBI in a multidisciplinary context. However, beyond support, if pharmacists fully engage in this service, they will also become accountable for the outcomes achieved. This is part of pharmaceutical care as defined by Hepler and Strand, which we fully endorse [38].

### Further research

This study brings a new approach to physical activity promotion in healthcare settings, by bringing together different healthcare professionals and sets a foundation to inform sectorial structures such as the national plan for physical activity promotion. Previous studies have resorted to consensus techniques to identify research priorities that can enable political change [39]. It seems clear that pharmacists are considered adequate promotors for physical activity, particularly by integrating their SBI with existing community structures. Further qualitative studies using in-depth interviews can identify more opportunities for interdisciplinary collaboration and interventions to be delivered across primary care structures. Our findings suggest that key success factors for implementation are education and training of pharmacists and interprofessional collaboration. Even though remuneration was not consensual, its absence may hinder feasibility given the conflicting demands in community pharmacies. However, as explained by Roger’s diffusion of innovation theory [40], not all pharmacies will be willing to accept this challenge simultaneously, thus implementation science is crucial to progressively generate evidence. These studies should in the future be supplemented with RCTs and cost-effectiveness approaches.

## Conclusion

This study provides an interdisciplinary perspective on how pharmacists can support patients in behaviour change. Our findings suggest that it is important to promote physical activity across the entire healthcare system. Pharmacists and pharmacies can be part of the solution. However, the current remuneration system hinders full implementation of innovative services in public health. There is a perceived need to include physical activity promotion in the political agenda to foster implementation.

### Supplementary Information

Below is the link to the electronic supplementary material.

Supplementary file1 (DOCX 15 kb)

Supplementary file2 (DOCX 19 kb)
