# Supplementary material for: The role of community pharmacists and pharmacies in physical activity promotion: an interdisciplinary e-Delphi study
Source: Int J Clin Pharm. 2024 Apr 25;46(4):947–56. doi: 10.1007/s11096-024-01731-z (PMC11286711; doi:10.1007/s11096-024-01731-z)
Supplement: Supplementary file 1 — Supplementary file1 (DOCX 15 kb) [file 11096_2024_1731_MOESM1_ESM.docx]

Supplementary file 1 - Survey questionnaire items

| **Group 1 - Importance of promoting physical activity in the community** |
| --- |
| [Promoting physical activity should be part of the role of all health professionals, especially those working in primary health care]. |
| [It is important that community health workers assess and record physical activity indicators such as number of steps, minutes of physical activity, intensity, and sedentary time, among others]. |
| [It is important that in the community health professionals provide brief physical activity advice to their users]. |
| [Physical activity promotion should be included as a pre graduate training module for different health professionals]. |
| [Physical activity promotion should be included as a post graduate training module for different health professionals]. |
| [There is a need to include physical activity promotion in the policy agenda of health promotion by health professionals] |
| **Group 2 - The pharmacist as a promoter of physical activity** |
| [Ordinance 97/2018 expands the services that pharmacies can provide and indicates that "Pharmacies may also promote campaigns and programmes for health literacy, disease prevention and promotion of healthy lifestyles." The role of the community pharmacist in promoting physical activity falls under this ordinance]. |
| [Some countries have a model of "health champions" who are individuals with specific training to advise people to adopt a healthier lifestyle. Pharmacists can adapt this model and be “health champions” in promoting physical activity in Portugal]. |
| [Decree-law 62/2016 indicates that "The Ministry of Health may contract with community pharmacies, in their areas of competence, the provision of public health intervention services framed in the priorities of health policy, namely programmes integrated with primary health care". The promotion of physical activity can be framed within the services described in the decree-law]. |
| [The National Plan for Physical Activity Promotion of the Directorate General of Health aims to "Train health professionals and promote structural and functional changes in order to generalize the promotion of physical activity in health services". Pharmacists need to have specific training on physical activity promotion to be able to competently provide this type of service]. |
| [There is a need for the regulator of the pharmacy profession to create a new specific competence, which is not yet legislated, for the promotion of physical activity by pharmacists]. |
| [The pharmacist may have an important role in referring users to places or resources in the community where they can be more physically active]. |
| [Individual physical activity promotion may be the most important role the pharmacist can play in promoting physical activity] |
| **Group 3 - Promoting physical activity in community pharmacies** |
| [Community pharmacies are suitable spaces for physical activity promotion]. |
| [Pharmacy should be remunerated for brief advice on physical activity by the NHS]. |
| [Pharmacy should be remunerated for brief advice on physical activity by users]. |
| [The current remuneration model focused on the sale of drugs and health products from pharmacies is a barrier to implementing these types of services in the future]. |
| [Pharmacies should be more involved in organising physical activity promotion activities, such as walking groups]. |
| [Pharmacies' computer systems should be able to record the physical activity levels of their users]. |
| [Similar to what already happens with nutrition consultations, pharmacies could also have a differentiated consultation with an exercise professional]. |
| [Within 10 years it is expected that pharmacies could be considered suitable venues for physical activity promotion]. |
| **Group 4 - Opportunities for pharmacists’ intervention** |
| Promoting physical activity in pharmacies can be especially important in people living with the following diseases: [Type 2 diabetes]. |
| Promoting physical activity in pharmacies can be especially important in people living with the following diseases: [Depressive disorder] |
| Promoting physical activity in pharmacies can be especially important in people living with the following diseases: [Musculoskeletal pain] |
| Promoting physical activity in pharmacies can be especially important in people living with the following diseases: [Anxiety disorders] |
| Promoting physical activity in pharmacies can be especially important in people living with the following diseases: [Metabolic syndrome] |
| Promoting physical activity in pharmacies can be especially important in people living with the following diseases: [Chronic Obstructive Pulmonary Disease] |
| Promoting physical activity in pharmacies can be especially important in people living with the following diseases: [Rheumatoid arthritis] |
| Promoting physical activity in pharmacies can be especially important in people living with the following diseases: [Angina] |
| Promoting physical activity in pharmacies can be especially important in people living with the following diseases: [Heart failure] |
| Promoting physical activity in pharmacies can be especially important for the following population groups: [Elderly (> 65 years)] |
| Promoting physical activity in pharmacies can be especially important for the following population groups: [Pregnant women] |
| Promoting physical activity in pharmacies can be especially important for the following population groups: [Adolescents] |
| Promoting physical activity in pharmacies can be especially important for the following population groups: [Smokers] |
| Promoting physical activity in pharmacies can be especially important for the following population groups: [Obese people (BMI > 30)] |
| Promoting physical activity in pharmacies can be especially important for the following population groups: [People living in social isolation] |
| Promoting physical activity in pharmacies can be especially important for the following population groups: [People at high risk of chronic disease (e.g. diabetes, people with risk factors for cardiovascular disease)] |
