# Supplementary material for: The role of community pharmacists and pharmacies in physical activity promotion: an interdisciplinary e-Delphi study
Source: Int J Clin Pharm. 2024 Apr 25;46(4):947–56. doi: 10.1007/s11096-024-01731-z (PMC11286711; doi:10.1007/s11096-024-01731-z)
Supplement: Supplementary file 2 — Supplementary file2 (DOCX 19 kb) [file 11096_2024_1731_MOESM2_ESM.docx]

Supplementary file 2 – Experts’ comments to both rounds

| **Group 1 - Importance of promoting physical activity in the community** |
| --- |
| ROUND 1   - Take into consideration that counselling should not focus on the problem but on the solution. - I agree that knowledge of physical activity for all health professionals is crucial in generic advice for a healthier lifestyle and for correct signposting to the most skilled professionals. - It is important to clarify which health professionals we are talking about and what is the understanding of "health professional". A multidisciplinary approach is important. |
| ROUND 2   - I maintain my previous comments. In the ones I have changed, it is in the sense of introducing a brief formative component, in my opinion, in the scope of signalling to the qualified professional and promotion of interdisciplinary work. - Regarding the 2nd topic, although it seems very important to me the assessment and recording of physical activity indicators by the community healthcare workers, the ideal is that this could be consulted and done in the user's electronic health record (to which the multidisciplinary team, with the user's permission, could have access), thus avoiding the multiplication of work and possible inconsistency of information. - The pharmacist should be a promoter of a healthier lifestyle, which includes the promotion of activity. Physical activity is only one of the pillars, and should be approached by the pharmacist in an integral way with other pillars such as nutrition, stress management, improving sleep quality, etc. |
| **Group 2 - The pharmacist as a promoter of physical activity** |
| ROUND 1   - The pharmacist can be an important professional in promoting individual physical activity, but he/she must receive adequate training for that. In Portugal I think this is still not the case, unfortunately. - I think pharmacists as a promoter in physical activity could be operationalised in various spheres, the issues being too broad to have an opinion. It should be signposting in a multidisciplinary team or even employer. I agree that it is promoter within awareness/literacy rather than intervention and prescription. Regarding 'health champions' I think all health professionals will be so in various capacities. From what I know of the concept, they have a broad knowledge to inspire positive cognitive-behavioural change by directing to the services needed. Their intervention is limited to explaining and connecting in a holistic way. For example, an exercise professional may indicate that this should be taken with awareness and information, referring to the professional who should give the informed resource to the user. - I understand promotion as actions, interventions or activities such as counselling/recommendation/incentive, provision of health literacy tools related to the topic, organisation of one-off or fixed activities, for example. |
| ROUND 2   - I stand by previous comments, adding that the rephrased sentence seems more appropriate, however even verbal this should, in my view, be general advice and referral to qualified professionals. The questions I have amended are along the same lines as the previous explanation. - I agree as long as you have basic knowledge is that you can guide the user in the best way to promotional of the area. |
| **Group 3 - Promoting physical activity in community pharmacies** |
| ROUND 1   - Professional work should be remunerated, by whom...it will depend on the context probably.... Ideally the NHS but we know that may not be a priority at present. If there was a general context of health promotion and physical activity in society since always these questions would not arise. - As for question 2 and 3 I can't figure out if it will be the pharmacy or the pharmacist. The pharmacy getting paid for the information by a qualified professional yes, by another professional no. Here I think it comes down to consumer law. I fully agree that the pharmacy is an establishment that is very close to the community, often the first point of contact and therefore a vehicle of choice for promoting healthy lifestyles. For this reason, as in other areas of health (nursing, nutrition), physical activity should be integrated in a multidisciplinary approach, in order to provide the best service to the user/customer. - When I mention 5 in questions 2 and 3, it is to state agreement with a shared cost model (similar to what happens with medicines), as there should be shared responsibility. - A brief intervention to promote physical activity can be framed in the interventions that pharmacists already develop and complement the counselling that is already done in the context of chronic disease management. In the current framework, I find it difficult to see this counselling being an independent service. |
| ROUND 2   - Questions 2 and 3 [about remuneration] - considering that this is a pharmaceutical consultation. - I stand by my previous comments, adding to the point I amended that it should include a qualified professional. - Similar to what happens with other services (e.g., nutrition provided by nutritionists), it makes sense that the pharmacy can provide a complementary service of support and prescription of physical activity (provided by a professional with evidence of competence). In relation to remuneration for the service, my response is that it makes perfect sense to exist if effectively a specialised, structured, quality service that presents health benefits is provided. - I think it's very important. Sometimes the pharmacy is the most trusted place in terms of health for the population. - I believe that brief advice on physical activity should be an integral part of the provision of information to the patient and therefore should not be remunerated. This type of information should be provided to the patient/patient/client at every contact that makes sense. Already a differentiated service in physical activity that includes a consultation should be remunerated. |
| **Group 4 - Opportunities of pharmacists’ intervention** |
| ROUND 1   - The promotion of physical activity in certain population groups or people with certain diseases should be done with great care and with caution about the risks inherent in inappropriate physical activity. - I think that, again, with an integrated and qualified team it will be important for all these populations. However, for me, again the issues are broad, and it is not clear whether: will a qualified professional in the pharmacy establishment be the prescriber of physical activity, and then I agree; or will the pharmacist be the prescriber of physical activity and then I do not agree. - A brief intervention, according to the literature, should not exceed 10 minutes. Although physical activity is beneficial in all the pathologies mentioned, a brief intervention in some diseases is not enough and may even constitute a risk. Therefore, it will depend on the diseased condition. There is a need to introduce interventions of another nature, of integral assessment, risk stratification, monitoring... In these cases, I think that the brief intervention of promoting physical activity is not adequate. People should be referred to the health services prepared to perform the assessment, surveillance and monitoring of these patients. - The promotion of physical activity is important for all population groups and for individuals living with the different pathologies mentioned above. However, it is essential that the pharmacist has the appropriate knowledge to correctly inform about what type of activity to promote according to the population group. - In this case it depends, if it is by consultation with a physiologist then I agree it will be beneficial for all conditions. - When assessing chronic disease risk one should separate the high risk of cardiovascular disease/evidence from the high risk of metabolic disease... these are very different things that should be treated differently. All the populations mentioned can benefit from increased physical activity but some are at higher risk than others and should be referred to an exercise professional to benefit from the most appropriate and safe approach for their condition. |
| ROUND 2   - I emphasise again that promoting physical activity is important for all population groups and for individuals living with the different pathologies mentioned above. However, it is fundamental that the pharmacist has the appropriate knowledge to correctly inform about what type of activity to promote according to the population group. |
